# Epidemic Cholera in Columbus, Ohio

**Published:** 1833

**Authors:** William McClay Awl

**Affiliations:** Columbus, Ohio


### Art. II.—Epidemic Cholera in Columbus.

*A brief history of the Epidemic Cholera as it appeared in the town of Columbus, and the Penitentiary of Ohio, in the summer of* 1833. By William M. Awl, M. D.

I am about to appear as the brief historian of our late attack of Epidemic Cholera, at the request of several correspondents and a number of personal friends. It would seem, moreover, to be the duty of every observer, to communicate whatever may be likely to assist the medical historian in completing a monograph of this all pervading pestilence. To avoid repeating what has been already published and republished elsewhere, I shall sum up what I have to say in as few words as possible.

Towards the close of June, the disease was reported to exist in the vicinity of Winchester, a small village 12 miles east of Columbus, on the Ohio Canal;—introduced into the settlement, it was reported and generally believed, by a stranger who had been left from a passing canal boat. By the mercy of Providence, it did not continue very long, nor spread very far through this unfortunate neighborhood. Nevertheless, the suffering was dreadful, both from disease and neglect, for a panic having seized the surrounding country, the affected spot was both proscribed and deserted, and most of those who sickened received little else than interment.

The first case among the citizens of Columbus, occurred on the 14th of July—it was under the care of a *Steam Doctor*, and proved fatal. This person, it was said, a short time before paid a visit to his Father, who resides in the *neighborhood* of the fatal district just mentioned, and hence a report and common belief, especially by those who consider the Cholera to be contagious, that the poison was conveyed into our town by this unfortunate individual. But his father lives 10 miles from that district, and it is not established that the deceased either saw or had communication with any one laboring under Cholera,—nor had he himself any connection with the second victim, who shortly afterwards took the disease in a different and distant part of the town. Besides, it is certainly true, that the occurrence of many unusually severe affections of the bowels, together with one or two very suspicious cases, had given ample warning of the destroyer's approach. With myself, therefore, and I believe most others, it is an established fact, that the distemper was unfolded simultaneously in different parts of the town, and that too independently of contagion.

For two weeks after its commencement, the epidemic was comparatively mild; spending its principal force among the convicts of the State Penitentiary; in which place it was severe, though of short duration, for after the first of August no new cases were reported.—There was a short respite, about the same time, for the citizens of the town,—when the usual intermittents made their appearance. These circumstances led the board of health to report favorably, and it was confidently believed, by the people, that the danger was over. But the whole proved to be a delusion, for the disease soon reappeared with redoubled force, in connexion with Fever, and during the two successive months, they raged in combination with great mortality.

Of the causes which seemed to favor this extraordinary duration of the epidemic—so plain and correct a statement is given, by a committee appointed of the board of health, that I cannot refrain from introducing it into this account.

*Final Report of the Board of Health.*—The undersigned, members of the Board of Health of the town of Columbus, being now happily released from the further performance of the duties of our appointment, by the restoration of general health, deem it a duty which we owe to the public to submit, in this our final report, a few general remarks in relation to the alarming disease which has recently afflicted our town.

The first case of Cholera occurred among us, on the 14th day of July, and the last on the 29th of September. Between these dates, the total number who fell victims to this disease within the limits of the town, was 92, of which 11 were among the prisoners confined in the Penitentiary. The total number of inhabitants in the town at the time the Cholera appeared among us, is supposed to have been about 3,500, of whom nearly one fourth part removed, temporarily, from the town, during the prevalence of the disease. It is worthy of remark that the Cholera continued in this place considerably longer than it has usually done in towns similarly situated, and comparing with it as to population. We have reason to believe that this may be attributed mainly to imprudent exposure; to improper living, both in regard to quality and quantity of food; and especially to the free use of highly stimulating medicines, recommended and taken under the mistaken belief, that they were antidotes against attacks of the Cholera. Indiscreet indulgencies in the use of fruit, and of crude, unripe or watery vegetables, are ascertained to have preceded in almost every instance, the attacks of the Cholera, and are believed to have had a direct and powerful agency in producing the disease. We are also fully satisfied that excess in eating and drinking was a prolific source of Cholera, and especially fever.

Of those who made free use of Cholera syrup, (a heating medicine recommended by a certain class of practitioners as a specific for the prevention of Cholera) a far greater portion were attacked with the disease, than of an equal number of persons who did not make use of it; and of those who were attacked after using this medicine in any considerable quantities, very few recovered. We have no doubt that a free use of this heating medicine was the immediate cause of many cases of obstinate fevers.

In support of these opinions, we refer to the fact, that in the Penitentiary, where strict rules in regard to diet were enforced, and where the use of heating remedies as means of prevention were prohibited, a very large proportion of those attacked with Cholera were cured, the disease was in a short time expelled, and the convicts subsequently enjoyed good health.

It is also true, that few among the temperate and regular livers, who avoided the use of the heating medicines recommended as preventives, were attacked either with Cholera or any other serious disease.

As some of the members of the Board of Health have been called upon by persons at a distance for information as to the success of the Thomsonian or Steam practice in the treatment of Cholera, we conceive it to be a duty which we owe to mankind, thus publicly to state as the result of experience in this place, and of our own observation, that no reliance can be placed on that system of practice, either for the prevention or cure of this formidable disease.

In the early part of the time, during which the Cholera prevailed among us, many of our citizens placed much reliance on the Thomsonian remedies. This confidence led them to disregard the precautions so generally recommended and deemed of so much importance in the prevention of Cholera, by the regular physicians. In several instances the premonitory symptoms were temporarily checked by the use of the Thomsonian remedy. This operated to increase the mistaken and fatal confidence which was reposed in these remedies, and in our opinion contributed greatly to increase the number and violence of the cases, and to prolong the continuance of the disease among us. These remedies only checked or disguised the symptom; they did not remove the cause of the disease. And when like a smothered fire, it broke forth with increased violence, fully developed, the remedies of that system were found in almost every instance, to be utterly powerless.

During the latter part of the time that the disease prevailed here, very few persons could be found who were willing to trust their lives or those of their friends in the hands of the Thomsonian practitioners: and the extraordinary mortality which occurred in the families of those who practiced physic on the Thomsonian plan, and among those who placed such entire reliance on their skill, sweeping off in several instances, nearly whole families, affords melancholy but strong evidence of the inefficiency and worthlessness of remedies in which such entire and fatal confidence has been placed. We are neither of us physicians; and in making these remarks, we disclaim all personal interest and feelings, as well as personal hostility towards those who practice on the Thomsonian system.

P. B. WILCOX,<br>

ALFRED KELLEY,<br>

J. PATTERSON,<br>

WM. MINER, } *Board of Health.*<br>

C. HEYL,<br>

J. BUTTLES,<br>

J. NOBLE.

Columbus, Nov. 2, 1833.

**Note.—It is to be understood that all manner of Botanical, Reformed and Steam-Doctors, are included under the name of Thompsonians in the above report, as they all consulted and practised together during the Cholera.**

Independent of a countless variety of real and imaginary minor complaints, the whole number of sick, during the continuance of the Cholera, may have amounted to between four and five hundred—of which, perhaps two hundred and fifty had actual Asiatic Cholera. The deaths from fever and other disorders, during the same period, were about sixty, including many young children. It is worthy of remark, that the temperate and prudent, and especially those who resided in comfortable circumstances, with good houses, very generally escaped. The Epidemic appeared to surround, as it were, the whole town,—to prey upon its circumference, while the central and principal street, suffered comparatively but in a very small degree. And in the adjoining village of Franklinton, west of the Scioto river, which contains some three or four hundred inhabitants, and is distant but one mile, there originated but two very slight cases, both of which terminated favorably.

By order of his excellency the Governor, I was selected as assistant physician to the State Penitentiary; and as the practice of Dr. Wright and myself proved generally successful, the following particulars in relation to that establishment, may not prove uninteresting.

The disease first appeared among the convicts on the 19th of July. Very few escaped without more or less disturbance in the bowels. One hundred were taken down with the premonitory symptoms and confined to the hospital, of whom forty had decided Cholera, and eleven died; there was not a single case of fever while the Cholera lasted. By the first day of August it ceased altogether, and before the 20th, the prison was reported to be in ordinary health. The whole number of criminals was two hundred and three. From seventy-five to a hundred were regularly detailed for out-door employment, on the erection of the new prison, exposed to the sun and weather; these were all retained within the walls after the sickness commenced, and the prosecution of the work for the time being suspended. A strict prohibition of every kind of vegetable was enforced, and the men were confined to the most temperate and rigid use of bread, ryecoffee and light soups, made principally of mutton. The buildings were frequently and thoroughly scrubbed and white-washed; and every room, together with the different entries, prison-yard, out-shops, gutters, privies, &c. &c. constantly sprinkled with the chloride of lime. Fumes from burning rosin and tar mixed with the atmosphere in every apartment, and a heavy bituminous smoke, from a half-smothered stone-coal fire, was permitted to envelope the out-shops and prison-yard on every morning. The beds and the bedding, both of the sick and the dead, were uniformly washed in hot soap-suds, and wrung out of a solution of the chloride of lime; and *be it remembered* that neither they who washed for the sick, or the attendants upon the diseased, the guards about the prison, the keeper or the physicians ever took the Cholera; and at one period, upwards of sixty men were confined, within touching distance of each other. Finally, the hospital department was freely ventilated, kept as clean and comfortable as possible, and well supplied with steady and faithful assistants, and the moment a man was known to be indisposed, he was ordered into it for examination, and, if necessary, placed under immediate treatment.

Of the eleven who died of the Cholera, nine had broken constitutions from previous intemperance, the 10th is said to have been sober, and the 11th occasionally took a drink, whenever he could get it. And what is not a little remarkable, the first nine drunkards had returned to punishment, for a second commission of crime.

In regard to general pathology, I have nothing to offer upon the subject of Cholera—*it is the Cholera*, and that seems to be the amount of what is as yet known concerning it—nor is it necessary for me to be minute in a regular detail of our treatment, more especially as we can add little or nothing to what is already before the public. Some practical remarks on a few of the various remedies, which have been tried for this disease, will, therefore, bring to a close this hastily written paper.

The *lancet* was seldom admissible in well formed Cholera. With a view of producing reaction, I am satisfied, from direct evidence, that it is utterly useless. But in the accompanying fevers, which of course wore the livery of Cholera, and were frequently characterized by distressing irritability of the stomach, and pains through the bowels, the loss of a few ounces of blood was quite indispensable, and occasionally the operation had to be repeated.

*Emetics* are highly important agents in the beginning of a case, particularly if the stomach is full, oppressed, or very sick, with chilliness, or distress about the head; or when the attack commences with severe cramps, more especially of the abdominal muscles. But their indiscriminate use was attended with some hazard, especially in rapid cases; and truth compels me to state, that I have undoubtedly known them hurry on the fatal stage of collapse.

*Epispastics* with me, were great favorites, particularly when applied to the epigastrium. If that region was tender, I always blistered it extensively, with as much expedition as possible; and I greatly prefer blisters to any other application that can be made to the same place; they not only remove the tenderness and irritability of the stomach, which are very seldom absent in this disease, but they rouse up the energies of the liver, and promote the salutary operation of medicines. Vesication is most speedily and effectually accomplished, by first covering the part with a sinapism, mixed up with equal parts of oil of turpentine and sharp vinegar. This plaster must be suffered to remain on, until the skin is perfectly reddened, and then the strong epispastic ointment is to be substituted. The blister should be from 10 to 12 inches square.

*Calomel* is the sheet anchor—the *sine qua non* in the treatment of Cholera; nor, if we except the blue pill, has it any substitute, *Lobelia and Bay-berry*, *Cayenne pepper and No.* 6, all to the contrary notwithstanding. It is true there are cases in which it fails—but every thing may and will fail in many cases of Cholera. Twenty-five or thirty grains of calomel, with about as many drops of laudanum, may be given at once, after which I prefer to repeat about the same quantity every two or three hours, until ninety or a hundred grains are taken; should it not operate in twelve or fourteen hours, castor oil or calcined magnesia may be administered, but there is no occasion for its hasty operation on the bowels; it will seldom salivate; and if, peradventure, such should be the case, there is an end to the disease, for the patient is cured. In proof of this statement, it is only necessary for me to mention the fact, that there occurred, from first to last—only one sore mouth in the prison, and the medicine was used in every case, in company with cold water and ice, for constant drinks. Every other remedy appears to be inferior to calomel. Warm applications and frictions, sinapisms, rubefacients and injections, are all proper, and may help to sustain life for a few miserable hours; but until this mercurial regulates the secretions and removes the blockade of the liver, so that bile can reappear in the evacuations, there never will be any genuine amendment.

*Astringent Injections*, when rice-water stools are copious and the patient appears to be sinking, should be instantly administered, and retained as long as it is possible. Unquestionably they are among the most useful and important of our remedies, to suspend the evacuations and save the fleeting strength. They should never be given warm, always *perfectly cold;* and, if necessary, repeated again and again, until they can be retained. Decoctions of oak bark, log wood, or gum kino, will answer the purpose. Perhaps the solution of sugar of lead, with the addition of a tea-spoonful of laudanum, will be found to succeed better.

*Camphor* may be occasionally useful, but all diffusible stimulants invariably do harm.

*The inspissated bile* of the ox, appeared to be useful in two cases, but manifested little power in subsequent trials. I used venous injections in one protracted case, without any benefit.

*Dry frictions* must be actively employed, to remove the clammy perspiration and restore the energies of the surface; but it is entirely unnecessary that the skin should be flayed off with improper roughness.

Time, vigilance and perseverance, are every thing in the treatment of Cholera; for if the case is abused or neglected, it hurries away to the state of collapse, for which there seems to be no remedy.

*Columbus, Ohio, November,* 1833.

